# Electrospun CNT embedded ZnO nanofiber based biosensor for electrochemical detection of Atrazine: a step closure to single molecule detection

**DOI:** 10.1038/s41378-019-0115-9

**Published:** 2020-01-13

**Authors:** Patta Supraja, Vikrant Singh, Siva Rama Krishna Vanjari, Shiv Govind Singh

**Affiliations:** 1 Department of Electrical Engineering, Indian Institute of Technology, Hyderabad, Telangana 502285 India; 2 School of Medicine, University of California Davis, Davis, California USA

**Keywords:** Electrical and electronic engineering, Carbon nanotubes and fullerenes, Biosensors, Environmental, health and safety issues

## Abstract

In this study we have reported the design and development of a facile, sensitive, selective, and label-free electrochemical sensing platform for the detection of atrazine based on MWCNT-embedded ZnO nanofibers. Electrospun nanofibers were characterized using scanning electron microscope (SEM), transmission electron microscope (TEM), X-ray diffraction (XRD), X-ray photoelectron spectroscope (XPS), UV-Visible spectroscope (UV-VIS), and Fourier-transform infrared spectroscope (FTIR). Electrochemical properties of MWCNT-ZnO nanofiber-modified electrodes were assessed using electrochemical impedance spectroscopy (EIS) and cyclic voltammetry (CV). Binding event of atrazine to anti-atrazine antibody, which immobilized on nanofiber-modified electrode via EDC and NHS chemistry, was transduced with EIS. Due to high conductivity, surface area, and low bandgap of MWCNT-ZnO nanofibers, we have achieved the sensitivity and limit of detection (LoD) of sensor as 21.61 (KΩ μg^−1^ mL^−1^) cm^−2^ and 5.368 zM for a wide detection range of 10 zM–1 µM. The proposed immunosensing platform has good stability, selectivity, repeatability, and reproducibility, and are less prone to interference.

## Biosensors: pesticide detection based on zinc oxide multi-walled carbon nanotubes

An electrochemical sensing platform has been developed for detecting the pesticide atrazine based on zinc oxide (ZnO) nanofibers (nanometer-scale fibers) embedded with multi-walled carbon nanotubes (MWCNTs). Carbon nanotubes are tubes of carbon with nanometer-scale diameters and MWCNTs consist of nested single-wall carbon nanotubes. Atrazine is a widely used pesticide with a long half-life and consumption of atrazine-containing water can result in severe health problems. However, previous methods for detecting atrazine have been limited by factors related to cost, time, size, and sample preparation. A team headed by Professor Shiv Govind Singh at the Indian Institute of Technology, India, succeeded in developing an extremely sensitive biosensor for detecting trace amounts of atrazine based on MWCNT-ZnO hybrid nanofibers. The authors believe that their sensing platform offers good stability, selectivity, repeatability, and reproducibility, and is stable against interference.

## Introduction

Pesticide is the mixture of organic substance used to prevent, destroy, or migrate any pest. In the agriculture sector crops, seeds are protected by pesticides. The cytotoxic and carcinogenic behavior of pesticides result in infertility, neurological disorders, and respiratory problems in humans. Usage of pesticides in fairly small areas can effect relatively larger areas by their movement though air/water or via seeping through soil. Among pesticides, the most broadly used are those from the chloro-triazine family. In particular, atrazine (1-chloro-3-ethylamino-5-isopropylamino-s-triazine; ATZ) is the most widely used pesticide of the triazine family, in crops due to its high efficiency^1,2^. In 1958, it was introduced as a herbicide for dicotyledons^3^. Due to biodegradation by microbes, the half-life time of ATZ in soil is 261 days, whereas degradation takes longer in water due to low solubility^4,5^. Consumption of ATZ-rich water causes several health problems such as endocrine disruption and hormone disruption, with the risk of breast and prostate cancer^6–8^. Notably, children are the most adversely effected. Further, exposure to ATZ during maternity period results in low fetal weight and limb/urinary/heart defects, with the risk of reduced survival on prolong exposure to high-level concentrations. According to the US environmental protection agency, the maximum acceptable level of ATZ concentration in drinking water is 3 parts per billion^9^, although long-term exposure to such low concentrations also effects the human endocrine system severely. On account of the adverse effects of ATZ on environment, it is desirable to develop biosensor platforms that can detect the same in water, both qualitatively and quantitatively.

Initially, several analytical techniques such as gas chromatography^10,11^, high-performance liquid chromatography^12–14^, capillary zone electrophoresis^15^, and gas chromatography coupled with mass spectroscopy^16,17^ have been employed for the detection of ATZ. Usage of these methods is limited on the basis of cost, time, miniaturization, and sample preparation. Also, these methods are not suitable for real-time, on-site in-situ detection of small molecules (molecular weight < 400 Da). On the contrary, immunosensors are the new-era device for on-site in-situ detection of molecules. Among several transduction methods used in immunosensing, such as optical^18,19^, electrochemical^20,21^, and piezoelectric transduction^22,23^ methods, electrochemical techniques are the most preferred due to their high sensitivity and fast response^24,25^. Also, for qualitative and quantitative detection of molecules, label-free electrochemical transduction mechanism is preferred over the labeled approaches. Furthermore, one can miniaturize the label-free electrochemical sensing platform into a biochip for plausible point-of-care applications^26–28^. While developing immunosensors, one needs to consider several important performance parameters, such as limit of detection (LoD), sensitivity, selectivity, and resolution, which inherently depend on the conductivity of transducing nanomaterial, surface area of the sensing electrode, functional modification of the sensing electrode, and binding ability of the desired antibody–antigen pair^29^. As understood, by carefully engineering the nanomaterials used for biotransduction, one can possibly achieve good sensitivity, resolution, and low LoD^30^. In view of this, in the present work, we have synthesized multi-walled carbon nanotube (MWCNT)-embedded ZnO nanofibers for label-free electrochemical detection of ATZ.

Recently, one-dimensional metal oxides have captured the attention of researchers in developing biosensors because of their inherent nanoscale chemical, electrical, and mechanical properties^31,32^. Among several metal oxides, ZnO has proved to be a highly promising material in developing biosensors in lieu of its unique properties such as high isoelectric point, high diffusion coefficient, good electron transferable capability, and biocompatibility^33–35^. However, being a high bandgap material, ZnO suffers from limited conductivity. Therefore, in view of improving its conductivity along with the surface area to immobilize antibodies, herein we have embedded ZnO nanofibers with MWCNT. Recently, MWCNTs grabbed the attention of researchers due to their high electrical conductivity, which can aid the bio-electrochemical properties for biosensors. Pure CNTs and/or their composites play a vital role as an electrode material in electrochemical applications because of unique properties such as high conductivity, nanotexture, and resiliency, and also they allow the nanoscopic dispersion of particles on the external nanotubular surface. In general, CNTs give an exceptional improvement of electrode performance due to their mesoporous and well-conducting networks. These conducting mesoporous networks accelerate charge transfer during the oxidation and reduction of reagents, as well as enabling a quick diffusion of reagents and reaction products. However, apart from mesopores that are perfect for ion transportation, accessible micropores are also required for the accumulation of ions in the electrical double layer. In general, for this, one demands a highly developed specific surface area. Therefore, pure CNTs as electrode materials can result in moderate electrochemical kinetics values (depending on their microtexture and catalyst impurities). Hence, to have a better electrochemical kinetics, CNTs need to have more defected outer walls along with embedding in heterogeneous atomic medium (polymers or metal oxides)^36^. In a previous work published by our group^37^, an MWCNT-ZnO nanofiber-based biosensor was reported for the detection of malarial parasites. The primary focus of the said work was to develop a label-free chemiresistive transduction scheme involving the nanomaterial for immunosensing. Further, the platform was realized on a flexible substrate. In contrast, the current work is aimed towards developing an electrochemical platform, which is an altogether different transduction scheme. Herein we analyze the material’s efficiency towards sensitive and selective detection of the target analyte in a typical three-electrode-based electrochemical cell.

**Fig. 1:**
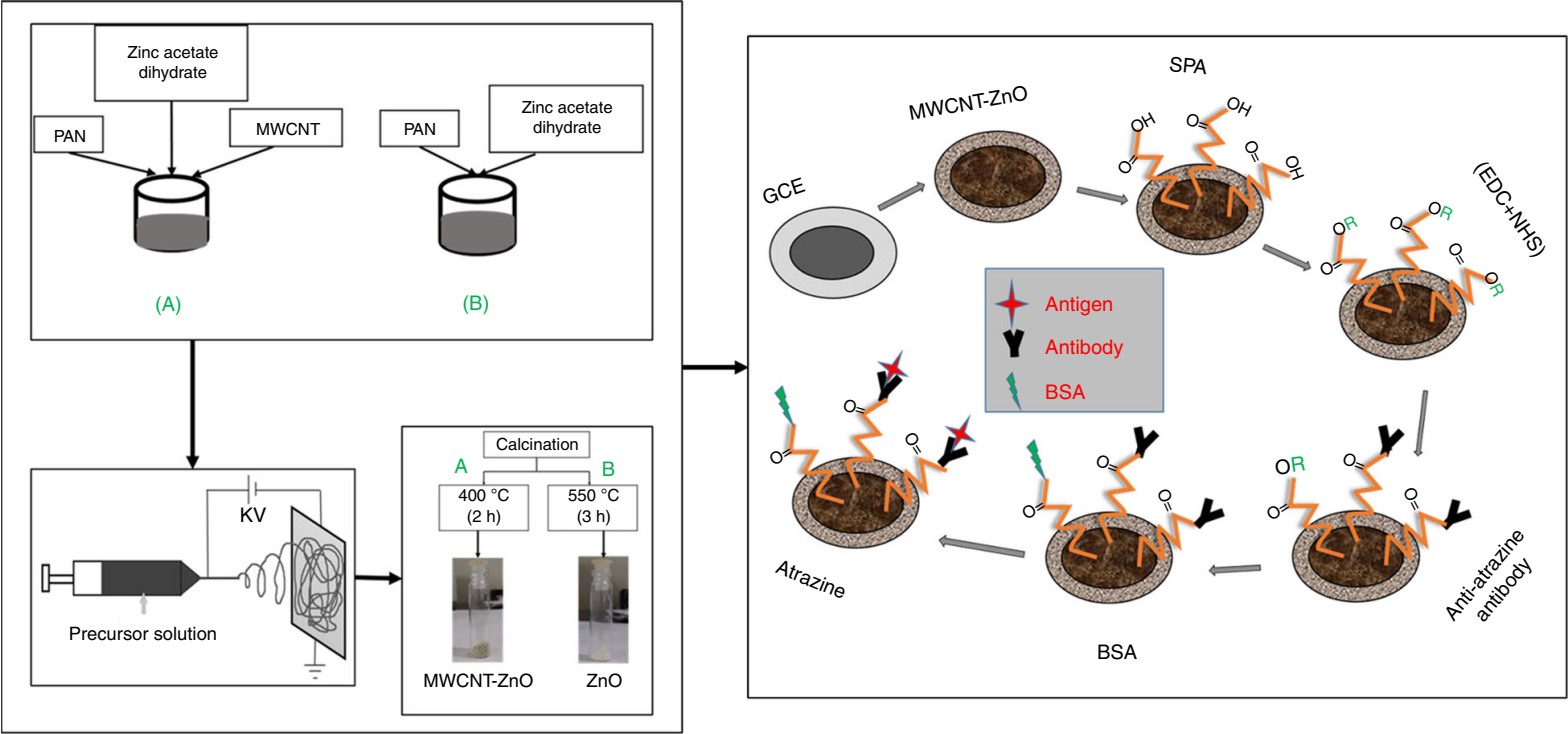
Step by step schematic representation of synthesis of MWCNT embedded ZnO nanofibers and preparation of bioelectrode. Schematic representation of proposed MWCNT-ZnO fiber-based biosensing platform for the detection of atrazine.

In this study, we are reporting an ultrasensitive label-free electrochemical transduction-based immunosensor for the detection of trace amounts of ATZ using MWCNT-ZnO hybrid nanofibers. The schematic representation of the proposed nano-biosensing platform for the detection of ATZ has been shown in Fig. 1. Furthermore, in Supplementary Table S6 we have presented a comparative analysis, wherein the performance of the proposed sensor is weighed against that of several previously reported literature. To the best of our knowledge, this is the best reported LoD among similar applications.

## Results

### Assesment of MWCNT-ZnO nanofiber characteristics

**Fig. 2:**
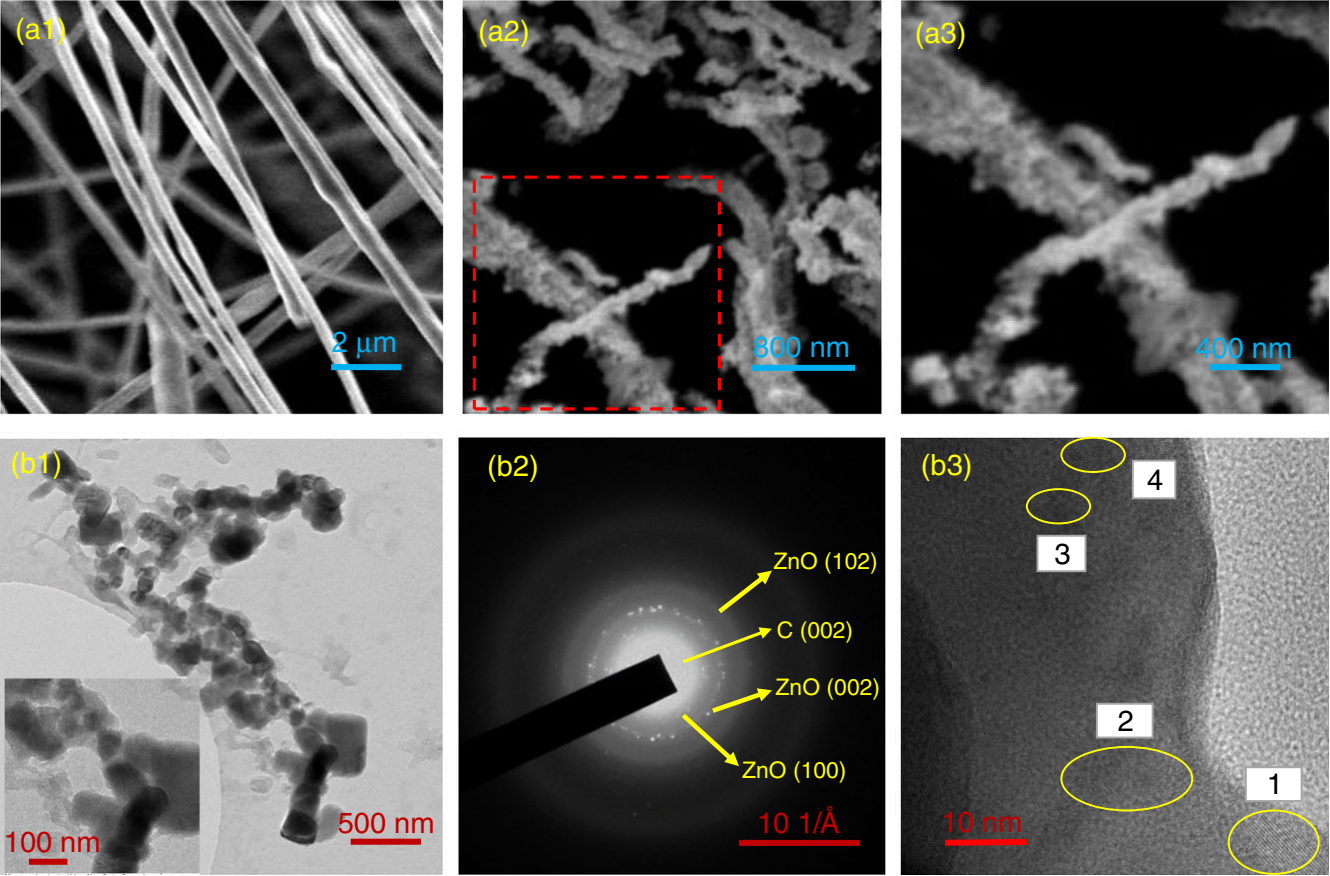
Morphological study of electrospun MWCNT embedded ZnO nanofibers using SEM and TEM. SEM images of MWCNT-ZnO nanofibers (**a1**) before calcination, (**a2**) after calcination at 400 °C, and (**a3**) high-resolution image of calcinated nanofiber. TEM images of MWCNT-ZnO nanofibers (**b1**) after calcination with an inset showing high-resolution magnified image (**b2**) SAED pattern and (**b3**) HRTEM showing an interface of polycrystalline MWCNT-ZnO nanofibers.

Morphological analysis of electrospun nanofibers, before and after the high-temperature calcination, was conducted using scanning electron microscope (SEM; proX, Phenom World) and transmission electron microscope (TEM; JEOL 2100). Figure 2a1 and a2 shows SEM images of MWCNT-ZnO nanofibers before and after calcination, respectively. Morphology of pre-calcinated nanofibers is smooth and continous with an average diameter in the range 700–900 nm. During calcination, the conducting polymer polyacrylonitrile (PAN) (carrier) decomposes at 400 °C, which inherently enhances the surface roughness of fibers, along with shrinkage in dimension. The diameter of post-calcinated fibers is in the range of 400–500 nm. Morphology of post-calcinated MWCNT-ZnO fibers in the magnified version has been shown in Fig. 2a3. The internal structure of MWCNT-ZnO nanofibers can be analyzed with TEM. Figure 2b1 shows the TEM image of MWCNT-ZnO nanofibers dropcasted on the copper grid, with the inset showing higher magnified focused image. The interface of nanofibers in calcinated nanofiber was observed with high-resolution TEM and are shown in Fig. 2b3. In addition, the selected area electron diffraction pattern of the polycrystalline MWCNT-ZnO nanofiber are shown in Fig. 2b2.

**Fig. 3:**
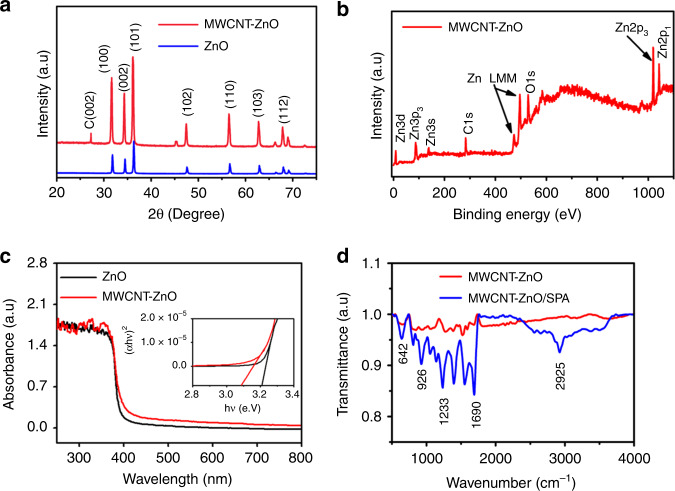
Characterizations of electrospun MWCNT-ZnO nanofibers. **a** XRD. **b** XPS. **c** UV-Visible. **d** FTIR.

Crystal structure and phase information of calcinated MWCNT-ZnO nanofibers was obtained from X-ray diffraction (XRD) measurements (PAN analytic X’ pert pro X-ray system with Cu Kα radiation, *λ* = 1.54 Å). To confirm the structural intactness of MWCNT in ZnO, we have plotted XRD peak information of ZnO and MWCNT-ZnO nanofibers on the same *x*-axis, as shown in Fig. 3a. The 2-Theta peaks at 31.5, 34.55, 36.27, 47.6, 56.5, 62.8, 66.29, 68.03, and 69.09 correspond to crystal planes (100), (002), (101), (102), (110), (103), (200), (112), and (201) respectively, of ZnO wurtzite structure. The extra peaks in the MWCNT-ZnO curve at 27.36 and 45.4 are due to the presence of graphite C(002) and C(100) planes, respectively. The observed peaks are well correlated with the literature^38–40^.

Elemental analysis of MWCNT-ZnO nanofibers was performed using X-ray photoelectron spectroscopy (XPS; ULVAC-PHI; Model pHI5000 Versa Probell). Figure 3b shows the XPS survey spectrum of MWCNT-ZnO nanofibers over a wide range of binding energies (0–1100 ev). Peaks at 1045.1 ev, 1020.1 ev, 86.63 ev, and 530.1 ev corresponds to Zn2*p*_1/2_, Zn2*p*_3/2_, Zn3*p*_3_, and O1*s*, respectively. Peak at binding energy 285.1 ev is due to the presence of *Sp*^2^ hybridized Carbon (C1*s*). Spectrum shown in Fig. 3b contains the peaks of zinc (Zn), oxygen (O), and carbon (C) elements only. The observed peaks are well correlated with the literature^41^.

The optical absorbance and bandgap of nanofibers were analyzed using UV-Visible spectroscopy (UV-VIS-NIR; PerkinElmer; Lambda-750). Figure 3c shows UV-VIS spectrum of ZnO and MWCNT-ZnO nanofibers at room temperature. The exciton band of ZnO nanofibers is observed at wavelength 388 nm, whereas for MWCNT-ZnO this is shifted towards a higher wavelength 415 nm. This shift in absorption peak to a higher wavelength is attributed to reduction in bandgap (*E*_g_ = *hc*/*λ*). We have calculated Bandgap *E*_g_ (*hν*) of synthesized MWCNT-ZnO and ZnO nanofibers as 3.09 eV and 3.21 eV, respectively, using Tauc relation. The detailed explanation of calculating the bandgap from UV-VIS absorbance spectrum has been given in Annexure A of the Supplementary Material. The calculated bandgap is well correlated with literature^42,43^.

To immobilize an anti-ATZ antibody on the nanofiber by chemisorption, one has to ensure the presence of a carboxylic functional group (–COOH) on the nanofiber after 3-sulfanylpropionoic acid (SPA) treatment. In view of this, Fourier-transform infrared spectroscopy (FTIR) (PerkinElmer; Model- Spectra 100) was carried out for MWCNT-ZnO nanofibers and SPA-treated MWCNT-ZnO nanofibers, and the spectrum are shown in Fig. 3d. Broad peaks in the range 2300–3500 cm^−1^ confirms the presence of –OH bond stretching. The existence of a single peak at 1690 cm^−1^ and multiple peaks at 1000–1500 cm^−1^ is due to –C = O stretching and –C–O stretching, respectively. Peaks at 642 and 432 cm^−1^ are characteristic to MWCNT-ZnO nanofibers. The presence of the above peaks in an FTIR spectrum of SPA-treated MWCNT-ZnO nanofibers ensures the surface carboxylic functionalization of nanofibers. The observed peaks are well correlated with literature^44,45^.

### Assessment of electrochemical properties of MWCNT-ZnO nanofibers

**Fig. 4:**
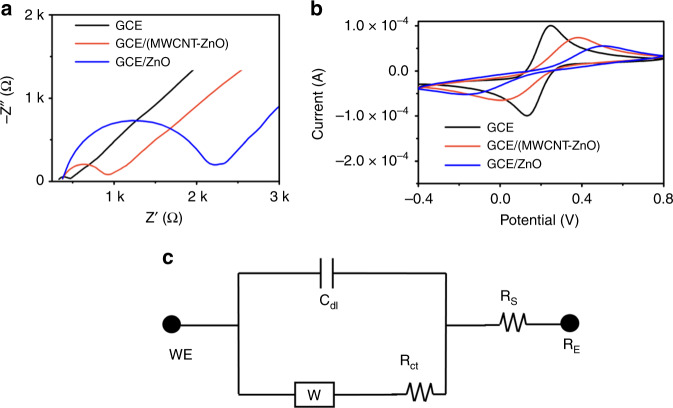
Electrochemical analysis of MWCNT-ZnO and ZnO nanofibers. **a** EIS. **b** CV. **c** Electrical modeling for EIS shown in **a**.

Electrochemical analysis of MWCNT-ZnO nanofibers modified was carried out using electrochemical impedance spectroscopy (EIS) and cyclic voltammetry (CV). Protocol for preparation of nanoofiber-modified electrodes has been mentioned in “Protocol for fabrication and electrochemical measurements of bioelectrode”. Figure 4a demonstrates the nyquist plot of bare and cleaned glassy carbon electrode (GCE), MWCNT-ZnO nanofiber-modified GCE (GCE/MWCNT-ZnO), and ZnO nanofiber-modified GCE (GCE/ZnO), and its equivalent electrical circuit has been modeled with Randles circuit and are presented in Fig. 4c. The extracted parameters of Randles circuit are shown in Annexure B of Supplementary Material. Here, the electrode performances can be compared on the basis of their charge transfer resistance (*R*_ct_), which is an indicator of rate kinetics. As seen, *R*_ct_ of ZnO nanofiber-modified electrode is more compared with that of bare GCE. This trend indirectly indicates the reduction in electron transfer kinetics at the electrode and electrolyte interface. To increase the rate kinetics, one has to improve the inherent conductivity of fiber. In view of this we have embedded MWCNTs in ZnO. As a proof-of-concept, we have verified the inherent conductivity of pristine ZnO and MWCNT-embedded ZnO nanofibers by direct *I*–*V* measurements and have plotted on the same voltage axis. The comparison plot is shown in Supplementary Fig. S4 in Annexure B of Supplementary Material. Protocol for the synthesis of MWCNT-ZnO nanofibers has been mentioned in “Synthesis of MWCNT-ZnO nanofibers.” Compared with GCE/ZnO, the charge transfer resistance of MWCNT-ZnO nanofiber-modified GCE is significantly high.

Figure 4b shows the cyclic voltammograms of GCE, GCE/MWCNT-ZnO, and GCE/ZnO electrodes, and electrochemical kinetics values of the above electrode’s CV, namely oxidative peak current (*I*_P_) and corresponding peak potential (*V*_P_), have been tabulated and are shown in Annexure B of Supplementary Material. The oxidative peak currents for GCE, GCE/MWCNT-ZnO, and GCE/ZnO are 99.9, 73.66, and 55.1 µA, respectively. Electrochemical characterization of GCE and GCE/MWCNT-ZnO-modified electrodes by varying scan rate from 20 to 100 mV s^−1^ has been presented in Supplementary Fig. S3 of Supplementary Material.

The effective surface area of nanofiber-modified electrode can be calculated by using Randles–Sevcik equation.

$$I_{\mathrm {p}} = 0.4463nFAC\left(\frac{{nFVD}}{{RT}}\right)^{0.5}$$

where *I*_P_, *n*, *F*, *A*, *C*, *V*, *D*, *R*, and *T* corresponds to the oxidative peak current of cyclic voltammogram, number of electrons transferred in redox event, surface area of electrode, concentration of nanofibers, scan rate, diffusion coefficient, universal gas constant, and temperature, respectively. At room temperature, Eqn (1) can be modified as below

$$I_{\mathrm {p}} = 268600\;ACD^{1/2}V^{1/2}n^{3/2}$$

Oxidative peak current of GCE/ZnO and GCE/MWCNT-ZnO electrodes is 55.1 µA (*I*_P1_) and 73.66 µA (*I*_P2_), respectively. Due to addition of MWCNTs to ZnO, the surface area of electrode (effective surface area) was increased by 33% compared with that of pure ZnO nanofiber-modified electrode. The detailed calculations of effective surface area of electrodes has been shown in Annexure C of the Supplementary Material. In conclusion, embedding of MWCNTs to ZnO enhanced the conductivity (indirectly *R*_ct_) and surface area (indirectly *I*_P_) of the electrode, compared with ZnO.

### Detection of atrazine

**Fig. 5:**
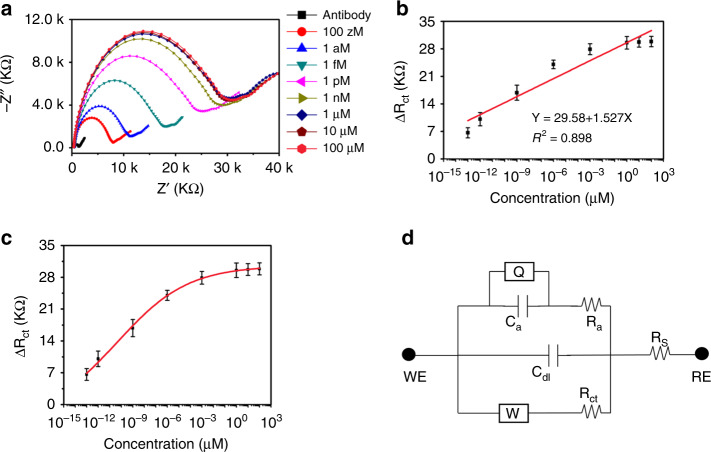
Electrochemical analysis of bioelectrode. **a** EIS analysis of anti-atrazine antibody-immobilized bioelectrode for various concentrations of atrazine, **b** calibration curve with linear fitting, **c** logistic sigmoidal curve fitting of data, and **d** electrical modeling of detection mechanism.

Electrochemical response of anti-ATZ antibody-immbolized bioelectrode for various concentrations of ATZ was tested with EIS instrument in [Fe (CN)_6_]^4−/3−^ spiked phosphate-buffered saline (PBS) (10 mM, pH 7.4) electrolyte solution, over the frequency range of 0.01 Hz–10 KHz. Initially known weight of ATZ was added to the carrier solvent and the resulting mixture was ultra-sonicated for 60 min to obtain a homogeneous 10 mg mL^−1^ concentrated aliquot. Later lower concentrations of ATZ was prepared through serial dilution protocol. Before the EIS measurement, we added 100 µL analyte-spiked PBS buffer to electrolyte and allowed the system to stabilize for 1000 s. During this period, ATZ molecules diffuse towards the surface of electrode and get attached to anti-ATZ antibody. Figure 5a shows nyquist plots obtained for a wide range of analyte (ATZ) concentrations 100 zM–100 µM. In view of comparing the performance of the proposed MWCNT-ZnO nanofiber-based sensing platform, we have tested the pure ZnO-based sensing platform for various concentrations of ATZ and EIS results are shown in Annexure D of Supplementary Material. Due to inherent nonconducting nature of ATZ, the binding event of ATZ to anti-ATZ antibody results in an increase of *R*_ct_. This is a result of the fact that electron needs to tunnel more distance at the interface of electrode/electrolyte, post the adsorption of ATZ on to the immobilized antibody. This results in reduction in electron transfer rate kinetics. As the traget concentration added to the solution increases, more number of binding events occur; as a result, one can observe further increment in *R*_ct_. Figure 5b shows the variation of *R*_ct_ with respect to antibody (Δ*R*_ct_ = *R*_ct_ − *R*_ct0_) for various concentrations of ATZ along with linear fitting of Δ*R*_ct_ values. Along with the linear fitting of data, we have plotted individual values of Δ*R*_ct_ against their ATZ concentration and have fitted with a logistic sigmoidal four-parameter curve. The fitting function is shown below

$$y = d2 + \frac{{(d1 - d2)}}{{1 + (\frac{x}{{x0}})^p}}$$

where *y*, *d*2, *d*1, *x*, *x*_0_, and *p* are change in charge transfer resistance (Δ*R*_ct_), maximum asymptote, minimum asymptote, concentration of analyte, concentration corresponding to inflation point (point at which 50% of the maximum signal change can be observed), and slope at the inflection point of the sigmoid, respectively. Sigmoidal fitting function for the curve shown in Fig. 5c is shown below. This calibrated function can be used to approximate the system response (Δ*R*_ct_) of sensor for any concentration of analyte (ATZ) closely, with an adjusted *R*^2^-value as 0.998.

$$y = 30.56 - \frac{{33.4}}{{1 + (\frac{x}{{4.71E - 11}})^{0.141}}}$$

To miniaturize the proposed sensing platform on to chip for point-of-care applications, one has to model the electrical behavior of the system. Figure 5d shows a modified Randles circuit, which was used to derive an equivalent model for the electrical behavior of sensing mechanism. In Fig. 4d, *R*_S_, *R*_ct_, *C*_dl_, *W*, *Q*, RE, and WE corresponds to solution resistance, charge transfer resistance, double layer capacitance, Warburg impedance, constant phase element, reference electrode (RE), and working electrode, respectively. Ra and Ca are the resistance and capacitance parameters, respectively, to model the binding adsorption phenomenon of ATZ. Parameters that were extracted from curve fitting of EIS graphs (MWCNT-ZnO nanofiber-based sensing platform) are shown in Annexure E of Supplementary Information. Sensitivity and LoD of proposed sensing platform were calculated using slope/(area of electrode) and 3.3*σ*/slope (*σ* is the SD of blank measurements), respectively. The proposed MWCNT-ZnO nanofiber-based electrochemical sensing platform accounts good sensitivity 21.61 (KΩ μg^−1^ mL^−1^) cm^−2^ for wide range of detection 100 zM–1 µM with LoD as 5.368 zM. Comparison of the proposed sensing platform with a reported literature has been shown in Annexure F of Supplementary Material.

### Repeatability, reproducibility, selectivity, stability, and interference analysis

Repeatability, reproducibility, interference, selectivity, and stability are the key factors needed to consider when determining the efficiency of a sensing platform. Protocol for testing the above parameters has been mentioned in “Protocol for testing repeatability, reproducibility, selectivity, stability, and interference.”

**Fig. 6:**
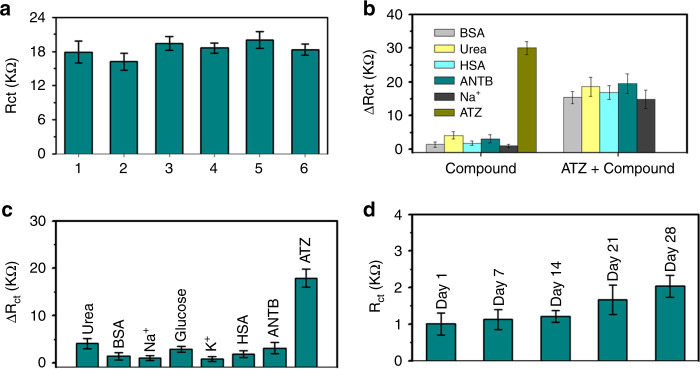
Study and evaluation of efficiency of proposed electrochemical sensing platform. **a** Study of reproducibility of six identical bioelectrodes for 1 fM of atrazine (*N* = 5). **b** Interference analysis: bar diagram representation of Δ*R*_ct_ for each 1 nM of pure nonspecific compounds (BSA, Urea, HAS, ANTB, Na^+^, Atrazine) and 1:1 mixture of atrazine and compound (*N* = 4). **c** Selectivity analysis: bar diagram representation of Δ*R*_ct_ for each 1 nM concentrated nonspecific compounds and 1 fM of atrazine (*N* = 4). **d** Stability analysis: bar diagram representation of *R*_ct_ for 28 days storage of bioelectrode (*N* = 4).

Figure 6a demonstrates the electrochemical response (*R*_ct_) of six identical bioelectrodes against 1 fM concentrated ATZ. The relative SD (% RSD) of six electrodes was calculated as 7.17%. The value of RSD shows that reproducibility of the proposed sensing platform is fairly good. The error bars of individual histograms shown in Fig. 6a corresponds to the SD of *R*_ct_ values obtained by measuring the electrochemical response of bioelectrode for five times with 180 s time gap between successive measurements. The maximum and minimum RSD obtained was 10.6% and 4.8% for electrode1 and electrode4, respectively. RSD of the remaining electrodes lies in between 10.6 and 4.8%. From the data provided in Fig. 6a, we can conclude that repeatability of proposed MWCNT-ZnO nanofiber-based sensing platform is good.

Stability of bioelectrode was tested by storing the anti-ATZ antibody-immobilized electrode at 4 °C for 28 days. Figure 6d shows the electrochemical response (*R*_ct_) of bioelectrodes, which was measured periodically every 7 days. *R*_ct_ values and associated error values of bioelectrodes after storage for 7, 14, 21, and 28 days are 1.124, 1.212, 1.664, and 2.04 KΩ, and 0.28, 0.16, 0.4, and 0.3, respectively. There was no significant change in values of *R*_ct_ observed even after storing for 28 days. This clearly indicated that anti-ATZ antibody-immobilized bioelectrode has good long-term stability.

Figure 6c demonstrates the selectivity of MWCNT-ZnO nanofiber-based sensing platform was against 1 nM concentrated nonspecific targets such as Na+, K+, bovine serum albumin (BSA), Glucose, human serum albumin (HSA), Urea, amoxicillin antibiotic (ANTB), and 1 fM specific target (ATZ). Change in *R*_ct_ with respect to blank (response of antibody-immobilized electrode without any analyte) was noted after every target addition and represented as histograms for better visual. Error bars associated with histograms corresponds to SD of measurements obtained from four identical electrodes (*N* = 4) and are presented in Annexure G of Supplementary Material. When compared with Δ*R*_ct_ of 1 fM ATZ, the recorded Δ*R*_ct_ values of 1 nM nonspecific targets was insignificant. From Fig. 6c, we can infer that even for high concentrations of nonspecific targets (six orders higher than ATZ concentration), the sensor response is poor, indicating high degree of selectivity.

Figure 6b shows the interference study of ATZ with compounds BSA, Urea, HSA, ANTB, Na+, and Glucose. As mentioned in “Protocol for testing repeatability, reproducibility, selectivity, stability, and interference,” initially we have recorded the response of bioelectrode for 1 nM dosage of interfering compounds. Subsequently, response (Δ*R*_ct_) for 1:1 mixture of ATZ and interfering compound was measured and represented as histograms in Fig. 6b. Error bars associated with histograms corresponds to SD of measurements obtained from four identical electrodes (*N* = 4) and are presented in Annexure G of Supplementary Material. From the information provided in Fig. 6b, we can infer that the proposed MWCNT-ZnO-based sensor was not affected adversely due to the presence of interfering compounds in testing medium.

## Discussion

Qualitative and quantitative detection of molecules whose molecular weight is <500 Da is inherently difficult because of their low molecular weight. Especially, molecules from the triazine family (ATZ) causes severe damage to flora and fauna even at low concentrations. Thus, it is highly essential to develop sensing platforms that can detect analytes ultra-sensitively at fairly low concentrations. Towards this, we have developed an electrochemical immunosensing platform with MWCNT-ZnO nanofibers. Obtained sensitivity and LoD of proposed sensing platform is 21.61 (KΩ μg^−1^ mL^−1^) cm^−2^ and 5.368 zM for wide detection range 10 zM–1 µM. In case of electrochemical immunosensors, low ranges of detection are often explained in terms of heterogeneity of the bioelectrode. Modeling of electrode–electrolyte interface is the most important in electrochemical sensors. In general, electron transfer rate kinetics is governed by the Butler Volmer’s equation (current density of electrode). This inherently depends on exchange current density and charge transfer coefficient (*α*). For low concentrations of analyte (added to the electrolyte), there was no significant change in surface concentration. As the bulk concentration is almost equal to surface concentration, the exchange current density almost remains constant. However, on the other hand, electrode current density exponentially depends on charge transfer coefficient, which inherently depends on activation barriers. Due to the nonconducting nature of ATZ, the binding event of ATZ to anti-ATZ antibody effects the activation barriers. As a result, the electrode current density decreases. This can be captured in terms of increment in charge transfer resistance (*R*_ct_) using EIS spectroscopy. In this point of view, heterogeneous electrodes give better electrochemical kinetic properties compared with homogeneous electrodes. In this work, we have fabricated the heterogeneous electrodes using MWCNT-ZnO nanofibers. Due to the modification of electrodes with MWCNT-ZnO nanofibers, we are able to increase the surface area of electrode by 33% compared with pure ZnO nanofibers. This improvement in surface area facilitates to immobilize more anti-ATZ antibodies on to the working electrode. Thus, the availability of binding sites for incoming ATZ molecule is more. Embedding of ZnO with MWCNTs also improved the conductivity of nanofibers compared with ZnO. We believe in this case, a combination of enhancement in conductivity and high surface heterogeneity (due to high surface area) is reasonable for a significant change in *R*_ct_ even at low concentrations of ATZ.

## Materials and methods

### Materials

For this work, *N*, *N*-dimethyl-formamide (DMF), zinc acetate dihydrate (Zn (CH_3_COO)_2_. 2H_2_O), PAN with average molecular weight 150,000 g/mol, BSA, HSA, SPA, *N*-hydroxysuccinimide (NHS), ANTB, urea, 1-Ethyl-3-(3-dimethylaminopropyl) carbodiimide (EDC), sodium chloride (NaCl), potassium chloride (KCl), Glucose, PBS tablets with pH 7.4, and ATZ were purchased from Sigma-Aldrich (USA). All the above chemicals were of analytical grade and were used as received without any further purification. Pristine MWCNTs with number of walls 3–15, length 1–10 µm, diameter 20–70 nm, and purity >95% was obtained from Reinste Nano ventures. Anti-ATZ antibody (ab30533) were purchased from Abcam (UK) vendors and stored at −20 °C. All glasswares used in this work were purchased from Hychem Laboratories. Deionized water with resistivity 18.4 MΩ cm^−^^1^ was obtained from Q-Millipore water purification system. Glassy carbon working electrode (GCE), Ag/AgCl RE, and platinum wire counter electrode (CE) were purchased from Sinsil International Mumbai.

### Methods/protocols

#### Synthesis of MWCNT-ZnO nanofibers

In this work, MWCNT-embedded ZnO nanofibers were synthesized by a well-known electrospinning method followed by high-temperature calcination. Initially, 4% (w/w) of MWCNTs were added to DMF (solvent) and were ultra-sonicated for 10 min followed by stirring for 30 min to get a homogeneous MWCNT solution (named as solution 1). Next, we have added 7% (w/w) of PAN (conducting polymer) to solution 1 and allowed to stir for 90 min at 60 °C (solution 2). Subsequently, 5% (w/w) of zinc acetate dihydrate (precursor) powder was added to solution 2. This composite mixture was allowed to stir for 210 min to obtain the required homogeneous precursor solution. As prepared, the precursor solution was loaded onto 5 mL syringe with 26-gauze needle without any air bubble. The solution was electrospun by applying 1.5 KV cm^−1^ electric field in between the tip of the metallic needle and grounded collector plate with a flow rate of 1 mL h^−1^. The flow rate and electric fields were optimized to form a stable Taylor cone, which can yield bead-free nanofibers. After 5 h of continuous spinning, fiber mats were collected from the plate and were calcinated at 400 °C for 180 min in a muffle furnance. This high-temperature calcination is necessary to remove PAN (carrier conducting polymer) and also to convert zinc acetate dihydrate to ZnO without decomposition of MWCNTs. To compare the MWCNT-ZnO with ZnO, we have synthesized the ZnO nanofibers by following the above procedure without MWCNTs.

#### Protocol for fabrication and electrochemical measurements of bioelectrode

In view of developing electrochemical biosensor for ultrasensitive detection of ATZ, one has to prepare the bioelectrode by immobilizing the anti-ATZ antibody on to the GCE with 3 mm diameter. This can be done through the formation of a peptide (CO–NH) bond. Initially, cleaned GCE was modified with MWCNT-ZnO nanofibers followed by carboxylic functionalization by dipping in SPA. Activation of carboxylic functional groups is done by treating with EDC-NHS; subsequently, the anti-ATZ antibody was immobilized via formation of a peptide bond. Deactivation of unbounded sites is done by using the blocker BSA. Protocol for fabrication of bioelectrodes has explained in detail in Annexure H of Supplementary Material. For better understanding, the schematic representation of fabrication bioelectrode is shown stepwise in Fig. 1.

All the electrochemical measurements reported in this study was carried out with CHI 660E electrochemical analyzer (CH Instruments, TX, USA). Platinum wire (CE), 3 mm diameter GCE (working electrode), and silver/silver chloride (RE) was used as electrodes of the electrochemical cell. PBS (10 mM, pH 7.4) spiked with 5 Mm potassium ferro/ferri cyanide ([Fe (CN)_6_]^4−/3−^) redox couple was used as an electrolyte. Modification in surface properties of bioelectrode upon adsorption of ATZ (target analyte) onto the anti-ATZ antibody yields change in the kinetics of reaction. This change in kinetics can be observed with EIS or CV. In view of modeling of biological event in electrical circuit form, we have chosen EIS. EIS studies were carried out with 5 mV ac input driving over a 0.184 V dc signal, in the frequency range 0.01 Hz to 10 KHz. CV measurements were performed in the voltage range of −0.4 V to 0.8 V, with a scan rate of 100 mV S^−1^ and sample interval of 1 mV.

#### Protocol for testing repeatability, reproducibility, selectivity, stability, and interference

The analytical performance of proposed sensing platform can be estimated by testing stability, repeatability, and reproducibility of bioelectrodes. In addition, selectivity towards nonspecific targets and their response to interference.

Repeatability of bioelectrode was tested by measuring the response of electrode for each concentration of ATZ five times with a short time gap (180 s) between successive measurements and are represented as error bars in calibration curves and histograms. Reproducibility of the sensor was tested by measuring separately the response of six different bioelectrodes for the same concentration of ATZ. Stability of antibody-immobilized bioelectrode was analyzed by storing it for 28 days at 4 °C and tested the performance periodically (7, 14, 21, and 28 days).

Selectivity of the proposed sensing platform was analyzed by measuring the response of anti-ATZ antibody-immobilized electrode for 1 ng mL^−1^ concentrated nonspecific targets such as urea, BSA, glucose, Na+, K+, HSA, and ANTB. Towards this, the aliquots of target reagents were prepared by adding 100 mg of the chemical in 1 mL of PBS, followed by serial dilution. One hundred microliters of nonspecific target was added to the electrolyte and the system was allowed to settle for 1000 s before taking the EIS measurement. We have tested the selectivity towards anti-ATZ antibody and cross-selectivity towards Beta Amyloid 1–42 antibody, and the results along with extracted parameters are presented in Annexure I of Supplementary Material.

To evaluate the efficiency of our proposed sensing platform, we also performed interference study. Initially, we measured the response of sensor for identical concentration (1 ng/mL) of BSA, HSA, Glucose, Na+, K+, and urea (interfering compounds). Subsequently, the biosensor’s response to a 1:1 mixture of ATZ and the interfering compound was measured.

## Supplementary information

Supplementary Material
