# Supplementary material for: Electrospun CNT embedded ZnO nanofiber based biosensor for electrochemical detection of Atrazine: a step closure to single molecule detection
Source: Microsyst Nanoeng. 2020 Jan 13;6:3. doi: 10.1038/s41378-019-0115-9 (PMC8433164; doi:10.1038/s41378-019-0115-9)
Supplement: Supplementary file 1 — Supplementary Material [file 41378_2019_115_MOESM1_ESM.pdf]

# Supplementary Material

# Electrospun CNT embedded ZnO nanofiber based biosensor for electrochemical detection of Atrazine: A step closure to single molecule detection

Patta Supraja, [1a] Vikrant Singh, [2b] Siva Rama Krishna Vanjari, [3a] and Shiv Govind Singh[*a]

[a] Department of electrical Engineering, Indian institute of Technology Hyderabad, Telangana, India 502285

[b] School of Medicine, University of California Davis, USA

[1] ee17resch01006@iith.ac.in

[2] vssingh@ucdavis.edu

[3]svanjari@iith.ac.in

[*]sgsingh@iith.ac.in

*Corresponding Author, Tel- 040-2302-6076, Fax- 040-2301-6032

## Annexure A

Optical bandgap of MWCNT-ZnO and ZnO was determined from UV-Vis absorption spectrum (absorbance vs wavelength) using Tauc relation.

$$\alpha h\nu = A\,[h\nu - E_g]^m$$

Where $\alpha$, h, $\nu$, A, $E_g$ and m corresponds to absorption coefficient, planks constant, frequency, constant, average bandgap and constant which depends on type of transition respectively. m is 0.5 for allowed direct bandgap.

$$\text{Absorption coefficient } \alpha = \frac{\text{Absorbance}}{\text{Thickness}}$$

Absorbance of 1µm thick sample was obtained from UV-VIS spectrum. For m=0.5 above equation was modified as below

$$(\alpha h\nu)^2 = h\nu - Eg$$

Above linear equation is in the form of Y= mX + C (where Y, X and C corresponds to $(\alpha h\nu)^2$, h$\nu$, and Eg respectively). For $\alpha$=0, Eg is equal to h$\nu$. In graphical point this optical bandgap (Eg = h$\nu$) can be determined as intercept of extended linear portion of $(\alpha h\nu)^2$ vs h$\nu$ plot on to the h$\nu$ axis. Figure S1 shows the $(\alpha h\nu)^2$ vs h$\nu$ plot with estimated bandgaps for MWCNT-ZnO and ZnO nanofibers.

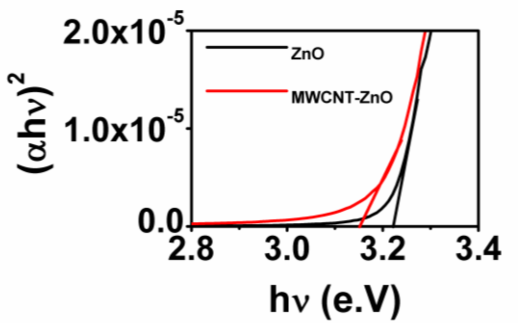


**Figure S1:** Tauc plot for MWCNT-ZnO and ZnO nanofibers

## TEM analysis of ZnO nanofibers:

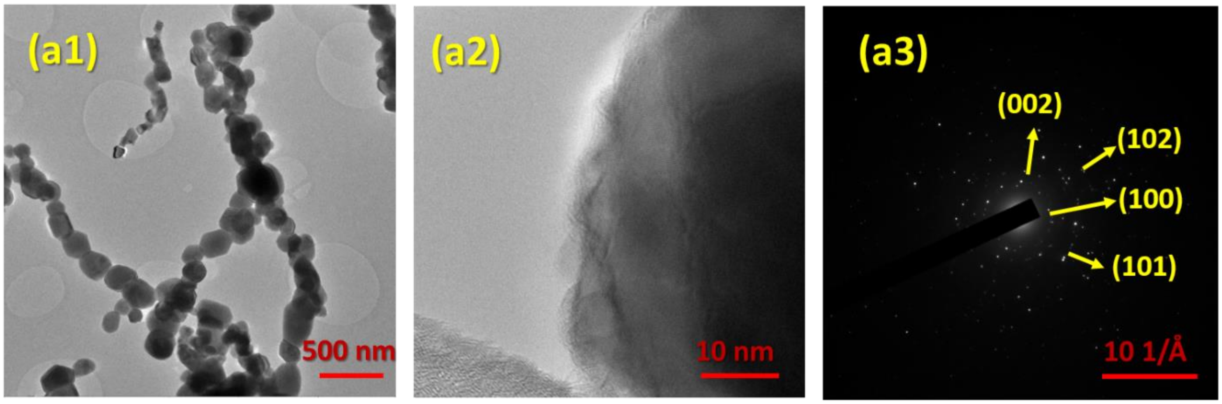


**Figure S2:** TEM images of ZnO nanofibers (b1) after calcination with inset high resolution magnified image (b2) HRTEM showing interface of polycrystalline ZnO nanofibers (b3) SAED pattern

## Annexure B

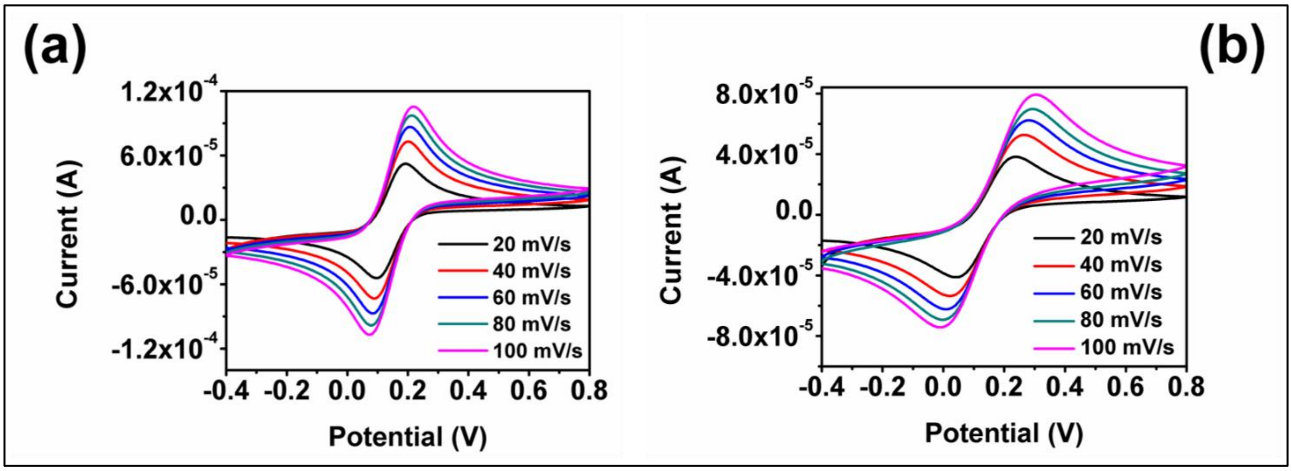


**Figure S3:** Electrochemical characterization of (a) *GCE (b) GCE/MWCNT-ZnO by varying scan rate from 20 mV/s to 100 mV/s.*

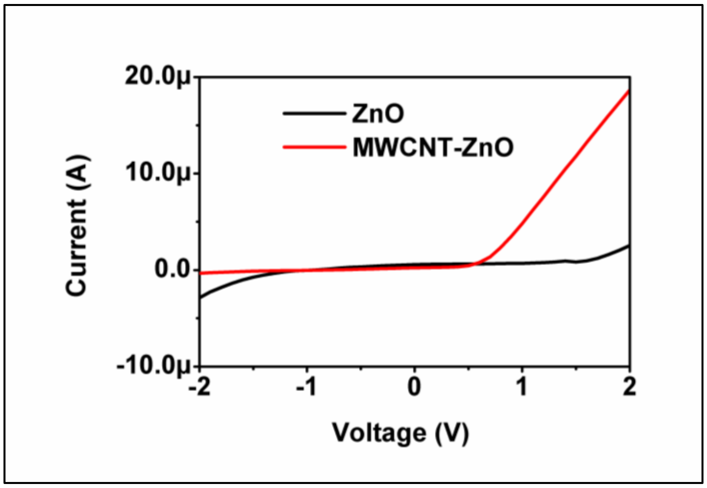


**Figure S4:** I-V characteristics of ZnO and MWCNT embedded ZnO nanofibers

**Table S1:** Randles circuit parameters extracted by curve fitting of Nyquist plot

| | $R_S$ (Ω) | Cdl (µF) | Rct (Ω) | W | Error (%) |
|---|---|---|---|---|---|
| **GCE** | 340.8 | 1.223 | 121.5 | 0.001105 | 3.9 |
| **GCE/ (MWCNT-ZnO)** | 398.6 | 1.097 | 486.2 | 0.001448 | 4.8 |
| **GCE/ZnO** | 405.3 | 1.093 | 1634 | 0.001504 | 5.1 |

**Table S2:** Electrochemical kinetic values extracted from CV of GCE, GCE/ (MWCNT-ZnO) and GCE/ZnO

| | $I_P$ (µA) | $V_P$ (Volts) |
|---|---|---|
| **GCE** | 99.9 | 0.2483 |
| **GCE/ (MWCNT-ZnO)** | 73.66 | 0.384 |
| **GCE/ZnO** | 55.1 | 0.513 |

## Annexure C

Effective surface area of electrode at room temperature can be calculated using Randles Sevcik equation.

$$Ip = 268600\ ACD^{1/2}V^{1/2}n^{3/2}$$

Where $I_P$, n, A, C, V, and D corresponds to oxidative peak current of cyclic voltagramm, number of electrons transffered in redox event, suface area of electrode, concentration of nanofibers, scan rate and diffusion coefficient respectively. Diffusion coefficient D was calculated using Ip (99.9 µA) of bare GCE (3 mm diameter). By substituting Ip, n, V, C and A as 9.9 µA, 1, 100 mv/s, 10 mM and $\Pi(1.5mm)^2$ in above equation we have calculated D as 1.662 x $10^{-6}$ $cm^2/s$.

The effective suface area of elctrodes after mofification with MWCNT-ZnO and ZnO nanofibers was calculated as 6.733 x $10^{-5}$ $cm^2$ and 5.03 x $10^{-5}$ $cm^2$ respectively, by substituting the parameters (mentioned in Table S1) in Randles Sevcik equation.

**Table S3**: Parameters to calculate effective surface area of ZnO and MWCNT-ZnO modified electrodes

| **Material/Parameter** | **ZnO** | **MWCNT-ZnO** |
|---|---|---|
| Ip (µA) | 55.1 | 73.66 |
| D ($cm^2/s$) | 1.662 x $10^{-6}$ | 1.662 x $10^{-6}$ |
| V (mv/s) | 100 | 100 |
| N | 1 | 1 |
| C (mM) | 10 | 10 |
| A ($cm^2$) | A1 | A2 |

## Annexure D:

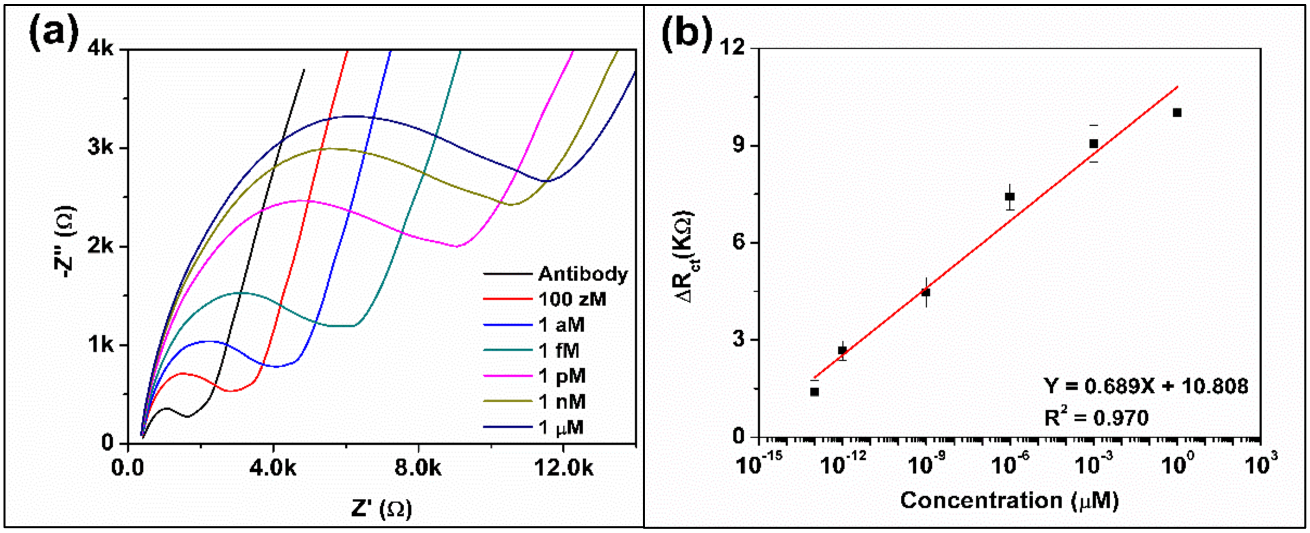


**Figure S5:** (a) Electrochemical Impedance Spectroscopic analysis of anti-atrazine antibody immobilized bioelectrode for various concentrations of atrazine using ZnO nanofibers (b) Calibration curve with linear fitting of change in charge transfer resistances

**Table S4:** Comparison of ZnO and MWCNT embedded ZnO nanofiber based atrazine sensing parameters

| **Material/Parameter** | **Sensitivity ($K\Omega/\mu g.mL^{-1}$)/$cm^2$** | **LoD (zM)** |
|---|---|---|
| **ZnO** | 9.75 | 11.9 |
| **MWCNT-ZnO** | 21.61 | 5.368 |

## Annexure E:

**Table S5:** Modified Randles circuit parameters extracted by curve fitting of Nyquist plot

| Parameter/ Concentration | $R_S$ (Ω) | Cdl (nF) | Rct (KΩ) | W | $R_a$ (Ω) | $C_a$ (nF) | Q (µ) | Error (%) |
|---|---|---|---|---|---|---|---|---|
| **Antibody** | 581.1 | 415.9 | 1.011 | 0.00145 | 1792 | 63.57 | 74.5 | 2.22 |
| **100 zM** | 353.2 | 228.8 | 7.785 | 0.00104 | 339.6 | 114.5 | 3.62 | 3.2 |
| **1 aM** | 497.8 | 202.1 | 11.24 | 0.0007445 | 247.9 | 158.5 | 3.707 | 2.2 |
| **1 Fm** | 475.5 | 243.2 | 18.11 | 0.0006075 | 388.3 | 126.7 | 3.011 | 1.08 |
| **1 pM** | 439 | 264.6 | 25.89 | 0.0003643 | 515.2 | 108.6 | 2.956 | 1.49 |
| **1 nM** | 438.4 | 268.9 | 29.99 | 0.0003125 | 591.9 | 98.75 | 2.519 | 1.57 |
| **1 µM** | 422 | 261.9 | 32.72 | 0.000426 | 451.6 | 102.1 | 2.78 | 2.77 |
| **10 µM** | 350.3 | 277.9 | 31.86 | 0.0003047 | 658.3 | 92.17 | 2.521 | 1.79 |
| **100 µM** | 390 | 275.1 | 31.89 | 0.0002794 | 689.7 | 97.66 | 2.451 | 1.39 |

# Annexure F

| Type of Detection | Immobilization Technique | Method of Detection | Detection limit | Reference |
|---|---|---|---|---|
| Label free | Bio functionalization of atrazine with 2d-BSA selectively on surface of the gold electrodes by thiol chemistry. *N*-acetylcysteamine to cover the gold electrodes | Electrochemical Impedance Spectroscopy | 40 ng $L^{-1}$ | [1] |
| Label free | Immobilization of antibody on to the gold electrode, which is modified with NTA substituted polypyrrole film. (polypyrrole NTA+Cu2 ions+ Antibody) | Cyclic Voltammetry and Electrochemical Impedance Spectroscopy | 10 pg $mL^{-1}$ | [2] |
| Label free | Coating of magnetic particles (magnetic monolayer) with streptavidin on to the gold electrodes. | Faradaic Impedance Spectroscopy and Cyclic Voltammetry | 10 ng $mL^{-1}$ | [3] |
| Surface Plasmon Resonance (SPR) | Covalent immobilization of analyte on gold coated electrode by alkanethiol self-assembled monolayer | SPR sensor system | 20 ng $L^{-1}$ | [4] |
| Labelled | Immobilization of monoclonal antibody on to the carbon electrode which is modified with Biodyne C membrane. Glucose oxidase which is labelled with HRP used for for Catalases and substrate scavenging. | Ampeoreometric SPE | 2 ng $L^{-1}$ | [5] |
| Labelled | Immobilization of HRP labelled single chain antibodies on to the screen printed electrode | Ampeoreometric SPE | 0.1 µg $L^{-1}$ | [6] |

| | | | | |
|---|---|---|---|---|
| | which is made up of polyaniline, polyvinyl sulfuric acid and carbon composite. | | | |
| Label free | Integration of molecularly imprinted nanofilms (MAPA+VIM+EGDMA) with SPR | AFM, ellipsometry and CA(Change in absorbance) | 0.091 nM | [7] |
| Optical fiber | Coating of 40nm thick Ag layer on unclad optical fiber followed by coating of molecular imprinted polymer with atrazine as template molecule | Spectral interrogation method<br>Shift in the resonance wavelength | 19.2 pM | [8] |
| Label free | Covalent immobilization of atrazine antibody through the functionalization of MWCNT-ZnO nanofibers by using SPA, (EDC-NHS), BSA. | Electrochemical Impedance Spectroscopy and Cyclic Voltammetry | 5.36 zM | This work |

**Table S6:** Comparison of proposed sensing platform with reported literature

## Annexure G

**Table S7:** Data of interference analysis along with error values (standard deviation)

| | **Compound** | | **ATZ + Compound** | |
|---|---|---|---|---|
| | **$\Delta R_{ct}$ (KΩ)** | **Error** | **$\Delta R_{ct}$ (KΩ)** | **Error** |
| BSA | 1.4 | 0.8 | 15.36 | 1.8 |
| Urea | 4.2 | 1.1 | 18.57 | 2.8 |
| HSA | 1.8 | 0.7 | 16.89 | 2.1 |
| ANTB | 3.1 | 1.2 | 19.49 | 2.9 |
| $Na^+$ | 1 | 0.5 | 14.84 | 2.8 |
| ATZ | 29.99 | 2 | - | - |

**Table S8:** Data of Selectivity analysis along with error values (standard deviation)

| **Compound** | **$\Delta R_{ct}$ (KΩ)** | **Error** |
|---|---|---|
| BSA | 1.4 | 0.8 |
| Urea | 4.2 | 1.1 |
| HSA | 1.8 | 0.7 |
| ANTB | 3.1 | 1.2 |
| $Na^+$ | 1 | 0.5 |
| Glucose | 2.9 | 0.6 |
| $K^+$ | 0.8 | 0.5 |
| ATZ | 29.99 | 2 |

# Annexure H

## Protocol for fabrication of bioelectrode

In view of developing electrochemical biosensor for ultrasensitive detection of atrazine one has to prepare the bioelectrode by immobilizing the anti-atrazine antibody on to the working electrode (GCE with 3 mm diameter). Initially GCE was polished with 0.05 micron and 0.1 micron alumina slurry subsequently rinsed with DI water several times and air dried at 60 $^{o}$C in incubator to get mirror finish surface. As cleaned GCEs were modified with MWCNT-ZnO nanofiber by drop casting 10 µL of 20 mg/mL MWCNT-ZnO solution and dried in incubator for 120 minutes at 60 $^{o}$C (GCE/MWCNT-ZnO). MWCNT-ZnO solution was prepared by ultrasonic dispersion of 20 mg of nanofiber in 1 mL of DMF solvent. In view of comparison, we also prepared ZnO nanofiber modified electrode (GCE/ZnO) by following above procedure with ZnO nanofibers dispersed solution. In order to immobilize antibodies onto the nanofiber one has to surface functionalize the nanofibers. This can be obtained by treating the nanofiber modified electrodes with SPA followed by (EDC-NHS). In detail, GCE/MWCNT-ZnO electrode was dipped in 10 Mm SPA and incubated at room temperature for 16 hours. This results in carboxylic functional groups (-COOH). Subsequently this –COOH functional groups were activated by drop casting 6 µL of EDC (coupling agent) and NHS (activator) mixture and incubated at room temperature for 4 hours. Next 6 µL of anti-atrazine antibody was drop casted and incubated at 4 $^{o}$C for 12 hours to form amide bonds (C-N) between ester group (-COOR) and amine (-$NH_2$) group of antibody. The unbounded sites were deactivated by dipping antibody immobilized electrode in BSA solution and incubated at 37 $^{o}$C for 30 min.

## Annexure I

Selectivity of proposed sensing platform towards anti-atrazine antibody for various concentrations of atrazine has tested with MWCNT-ZnO nanofiber modified GCE (GCE/(MWCNT-ZnO). Figure S1 (a) demonstrates the EIS responses of GCE/ (MWCNT-ZnO) for 1 pM, 1 nM, 1 µM and 10 µM of atrazine without anti-atrazine antibody. Change in $R_{ct}$ with respect to blank (response of MWCNT-ZnO nanofiber modified electrode) was noted after every target addition and represented as histograms for better visual in figure S1 (b). For better understanding we have compared the above results with the results obtained by adding the 1 pM, 1 nM, 1 µM and 10 µM of atrazine to the anti-atrazine antibody immobilized bioelectrode.

Similarly, cross selectivity of proposed sensing platform towards non-specific target has tested by immobilizing Beta-Amyloid 1-42 antibody (**AB(1-42) antibody**) on to the MWCNT-ZnO nanofiber modified GCE. Figure S1 (c) shows the EIS responses of AB (1-42) antibody immobilized electrode for 1 pM, 1 nM, 1 µM and 10 µM of atrazine. Change in $R_{ct}$ with respect to AB (1-42) antibody was noted after every target addition and represented as histograms for better visual in figure S1 (d). Table S6 and table S7 shows modified Randles circuit parameters extracted by curve fitting of Nyquist plots in Figure S1 (a) and Figure S1 (c) respectively. From results we can infer that even for high concentrations of atrazine the response of electrodes for non-specific antibody as well as without anti-atrazine antibody as well as very poor. This indicates that the proposed sensing platform has high degree of selectivity.

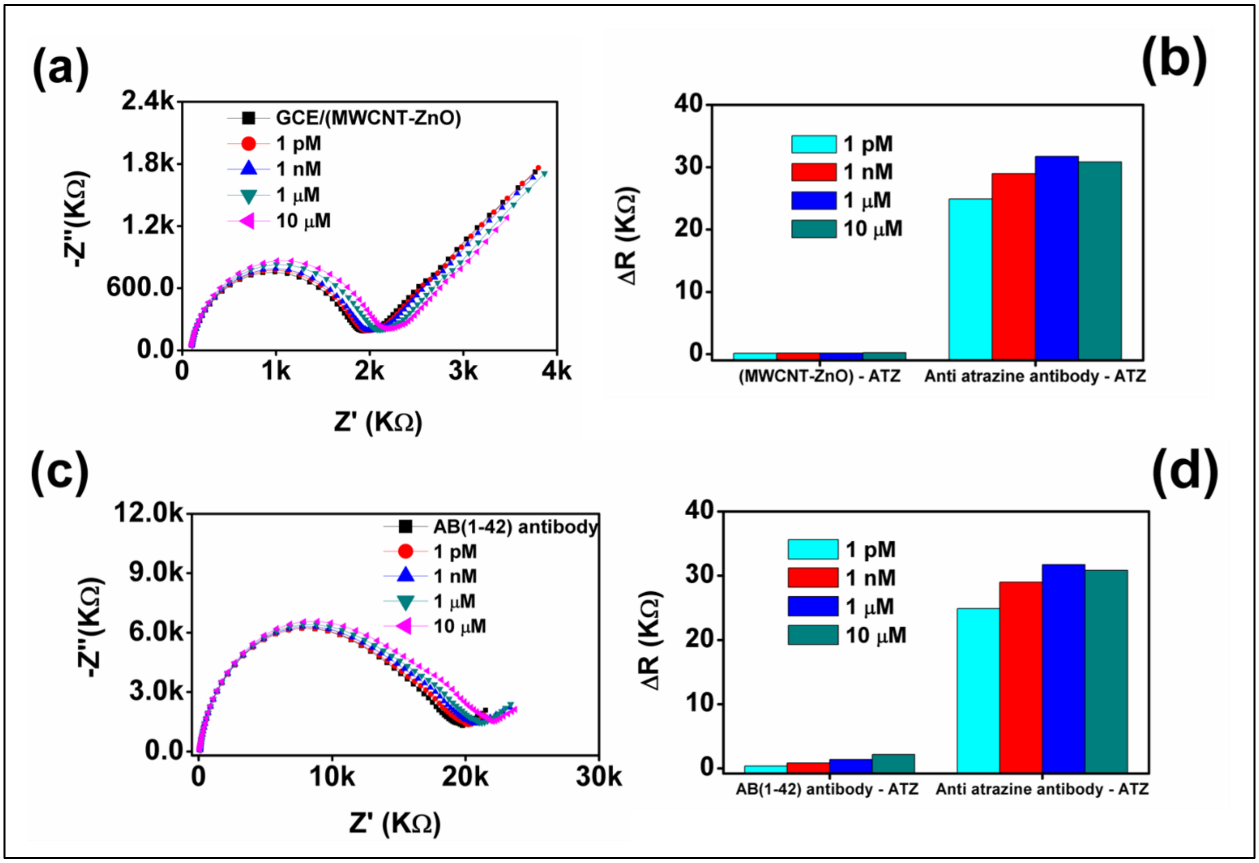


**Figure S6:** (a) EIS responses of MWCNT-ZnO nanofiber modified electrode for 1 pM, 1 nM, 1 μM and 10 μM of atrazine without anti-atrazine antibody (b) Comparison of $\Delta R_{ct}$ for various concentrations of atrazine with and without anti-atrazine antibody (c) EIS responses of AB (1-42) antibody immobilized bioelectrode for 1 pM, 1 nM, 1 μM and 10 μM of atrazine (d) Comparison of $\Delta R_{ct}$ for various concentrations of atrazine with anti-atrazine antibody and AB (1-42) antibody.

**Table S9:** Modified Randles circuit parameters extracted by curve fitting of Nyquist plots in Figure S6 (a)

| Parameter/ Concentration | Rs (Ω) | Cdl (nF) | Rct (KΩ) | W | $R_a$ (Ω) | $C_a$ (nF) | Q (μ) | Error (%) |
|---|---|---|---|---|---|---|---|---|
| **GCE/ MWCNT-ZnO** | 98.86 | 390.1 | 1.821 | 0.001311 | 310.4 | 105.1 | 105.1 | 1.16 |
| **1 pM** | 102.9 | 449 | 1.944 | 0.001472 | 323.6 | 102.4 | 102.4 | 1.79 |
| **1 nM** | 102.2 | 449.1 | 1.977 | 0.001245 | 387.4 | 98.9 | 98.9 | 1.8 |
| **1 μM** | 95.91 | 404.7 | 1.981 | 0.001228 | 451.6 | 95.5 | 95.5 | 1.18 |
| **10 μM** | 95.55 | 406.8 | 2.067 | 0.001227 | 423.9 | 94.49 | 94.49 | 1.01 |

**Table S10:** Modified Randles circuit parameters extracted by curve fitting of Nyquist plots in Figure S6 (c)

| Parameter/ Concentration | Rs (Ω) | Cdl (nF) | Rct (KΩ) | W | $R_a$ (Ω) | $C_a$ (nF) | Q (μ) | Error (%) |
|---|---|---|---|---|---|---|---|---|
| **GCE/ MWCNT-ZnO/AB 1-42 antibody** | 95.83 | 241.9 | 19.45 | 0.001192 | 918.6 | 58.9 | 3.23 | 1.13 |
| **1 pM** | 95.08 | 238.6 | 19.82 | 0.001133 | 825.6 | 56.45 | 3.33 | 1.34 |
| **1 nM** | 94.51 | 236.9 | 20.28 | 0.0009593 | 789 | 54.41 | 3.36 | 1.73 |
| **1 μM** | 95.49 | 235.5 | 20.82 | 0.001072 | 761.2 | 54.57 | 3.38 | 1.54 |
| **10 μM** | 94.72 | 232.7 | 21.59 | 0.001012 | 706.2 | 54.72 | 3.41 | 1.64 |

## References


[1] Ramón-Azcón, Javier, Enrique Valera, Ángel Rodríguez, Alejandro Barranco, Begoña Alfaro, Francisco Sanchez-Baeza, and M-Pilar Marco. "An impedimetric immunosensor based on interdigitated microelectrodes (IDμE) for the determination of atrazine residues in food samples." *Biosensors and Bioelectronics* 23, no. 9 (2008): 1367-1373.

[2] Ionescu, Rodica E., Chantal Gondran, Laurent Bouffier, Nicole Jaffrezic-Renault, Claude Martelet, and Serge Cosnier. "Label-free impedimetric immunosensor for sensitive detection of atrazine." *Electrochimica Acta* 55, no. 21 (2010): 6228-6232.

[3] Helali, Saloua, Claude Martelet, Adnane Abdelghani, Mhamed Ali Maaref, and Nicole Jaffrezic-Renault. "A disposable immunomagnetic electrochemical sensor based on functionalised magnetic beads on gold surface for the detection of atrazine." *Electrochimica Acta* 51, no. 24 (2006): 5182-5186.

[4] Farré, Marinella, Elena Martínez, Javier Ramón, Alicia Navarro, Jelena Radjenovic, Elba Mauriz, Laura Lechuga, M. Pilar Marco, and Damià Barceló. "Part per trillion determination of atrazine in natural water samples by a surface plasmon resonance immunosensor." *Analytical and Bioanalytical Chemistry* 388, no. 1 (2007): 207-214.

[5] Keay, R. W., and C. J. McNeil. "Separation-free electrochemical immunosensor for rapid determination of atrazine." *Biosensors and Bioelectronics* 13, no. 9 (1998): 963-970.

[6] Grennan, Kathleen, Gillian Strachan, Andrew J. Porter, Anthony J. Killard, and Malcolm R. Smyth. "Atrazine analysis using an amperometric immunosensor based on single-chain antibody fragments and regeneration-free multi-calibrant measurement." *Analytica Chimica Acta* 500, no. 1-2 (2003): 287-298.

[7] Saylan, Yeşeren, Semra Akgönüllü, Duygu Çimen, Ali Derazshamshir, Nilay Bereli, Fatma Yılmaz, and Adil Denizli. "Development of surface plasmon resonance sensors based on molecularly imprinted nanofilms for sensitive and selective detection of pesticides." *Sensors and Actuators B: Chemical*241 (2017): 446-454.

[8] Agrawal, Harshit, Anand M. Shrivastav, and Banshi D. Gupta. "Surface plasmon resonance based optical fiber sensor for atrazine detection using molecular imprinting technique." *Sensors and Actuators B: Chemical* 227 (2016): 204-211.
